# Distant recurrence in the cerebellar dentate nucleus through the dentato-rubro-thalamo-cortical pathway in supratentorial glioma cases

**DOI:** 10.1007/s00701-024-05981-8

**Published:** 2024-02-14

**Authors:** Masayuki Kanamori, Yohei Morishita, Yoshiteru Shimoda, Eiko Yamamori, Shiho Sato, Yoshinari Osada, Shin-Ichiro Osawa, Ichiyo Shibahara, Ryuta Saito, Yukihiko Sonoda, Toshihiro Kumabe, Hidenori Endo

**Affiliations:** 1 Department of Neurosurgery, Tohoku University Graduate School of Medicine, Sendai, Japan; 2 Department of Diagnostic Radiology, Tohoku University Graduate School of Medicine, Sendai, Japan; 3 Department of Neurosurgery, Kitasato University Graduate School of Medicine, Kanagawa, Japan; 4 Department of Neurosurgery, Nagoya University Graduate School of Medicine, Nagoya, Japan; 5 Department of Neurosurgery, Yamagata University Graduate School of Medicine, Yamagata, Japan

**Keywords:** Supratentorial glioma, Distant recurrence, Dentate nucleus, Dentato-rubro-thalamo-cortical pathway

## Abstract

**Background:**

Distant recurrence can occur by infiltration along white matter tracts or dissemination through the cerebrospinal fluid (CSF). This study aimed to clarify the clinical features and mechanisms of recurrence in the dentate nucleus (DN) in patients with supratentorial gliomas. Based on the review of our patients, we verified the hypothesis that distant DN recurrence from a supratentorial lesion occurs through the dentato-rubro-thalamo-cortical (DRTC) pathway.

**Methods:**

A total of 380 patients with supratentorial astrocytoma, isocitrate dehydrogenase (*IDH*)-mutant (astrocytoma), oligodendroglioma, *IDH* mutant and 1p/19q-codeleted (oligodendroglioma), glioblastoma, *IDH*-wild type (GB), and thalamic diffuse midline glioma, H3 K27-altered (DMG), who underwent tumor resection at our department from 2009 to 2022 were included in this study. Recurrence patterns were reviewed. Additionally, clinical features and magnetic resonance imaging findings before treatment, at the appearance of an abnormal signal, and at further progression due to delayed diagnosis or after salvage treatment of cases with recurrence in the DN were reviewed.

**Results:**

Of the 380 patients, 8 (2.1%) had first recurrence in the DN, 3 were asymptomatic when abnormal signals appeared, and 5 were diagnosed within one month after the onset of symptoms. Recurrence in the DN developed in 8 (7.4%) of 108 cases of astrocytoma, GB, or DMG at the frontal lobe or thalamus, whereas no other histological types or sites showed recurrence in the DN. At the time of the appearance of abnormal signals, a diffuse lesion developed at the hilus of the DN. The patterns of further progression showed that the lesions extended to the superior cerebellar peduncle, tectum, tegmentum, red nucleus, thalamus, and internal capsule along the DRTC pathway.

**Conclusion:**

Distant recurrence along the DRTC pathway is not rare in astrocytomas, GB, or DMG at the frontal lobe or thalamus. Recurrence in the DN developed as a result of the infiltration of tumor cells through the DRTC pathway, not dissemination through the CSF.

**Supplementary Information:**

The online version contains supplementary material available at 10.1007/s00701-024-05981-8.

## Background

Adult diffuse gliomas and diffuse midline gliomas, H3 K27-altered (DMG) are infiltrative tumors [20]. They occasionally recur at distant sites, including the cerebellum, brainstem, or intramedullary region of the spinal cord, and this distant recurrence is associated with poor prognosis [1, 8, 12, 14, 29, 36]. Therefore, attention should be paid to the vicinity of the resection cavity and the entire central nervous system. This distant recurrence may be attributed to leptomeningeal dissemination (LMD) through the cerebrospinal fluid (CSF) and infiltration along the white matter tracts [5, 6, 32]. Examples of infiltration along the white matter tracts include extension to the contralateral hemisphere via the corpus callosum [27], which is called “butterfly glioblastoma,” extension to the contralateral cerebrum via the anterior commissure [19], and extension to the brainstem via the corticopontine tract [5].

Distant recurrence can develop in the cerebellum [4, 5, 7, 10]. This type of recurrence has been considered to occur due to dissemination through the CSF [4, 7, 12, 14]. However, no reports have demonstrated a correlation between cytological findings and cerebellar recurrence. Cerebellar recurrence frequently develops at the hilus of the cerebellar dentate nucleus (DN) [4, 7, 12, 14]. Based on these findings, we hypothesize that distant cerebellar recurrence from a supratentorial lesion occurs through the white matter tracts that connect the cerebellar DN with the cerebrum and thalamus. The cerebellum and cerebrum are connected by networks with feed-forward and feed-backward connections, and these networks play an essential role in the execution of voluntary movements, motor planning, and neurocognitive functions such as abstract thinking and working memory [9, 22, 34]. The dentato-rubro-thalamo-cortical (DRTC) pathway [3, 9, 11, 15, 21] and the cortico-ponto-cerebellar pathway [5] are the major efferent pathway from the cerebellum to the cerebrum and the afferent pathway from the cerebrum to the cerebellum, respectively. The DRTC pathway originates from the DN and the emboliform, globose, and fastigial nuclei; courses through the superior cerebellar peduncle (SCP), contralateral red nucleus (RN), and ventrolateral or ventroanterior nucleus of the thalamus; and projects to the frontal lobe [9, 11, 24]. The cortico-ponto-cerebellar pathway runs from the cerebral cortex to the pontine nucleus and contralateral middle cerebellar peduncle and projects to the cerebellar cortex.

This study aimed to review cases of supratentorial gliomas with initial recurrence in the DN to clarify the clinical features and pathogenesis of recurrence based on the progression pattern.

## Methods

### Patients

This was a retrospective study reviewing the medical records of patients with supratentorial adult diffuse gliomas and thalamic DMG who underwent debulking surgeries at our department between January 2009 and December 2022 [12, 13, 16, 26, 29, 31]. The follow-up data until June 2023 were analyzed. The patients were diagnosed according to the World Health Organization classification of central nervous system tumors, fifth edition [20], which classified adult diffuse gliomas into astrocytoma, isocitrate dehydrogenase (*IDH*)-mutant (astrocytoma), oligodendroglioma, *IDH* mutant and 1p/19q-codeleted (oligodendroglioma), and glioblastoma, *IDH*-wild type (GB). This study was approved by the Institutional Review Board of Tohoku University Hospital (2021–1-393). Participants were given the option to opt out of this study.

### Estimation of recurrence on magnetic resonance images

According to the response assessment criteria in neuro-oncology, recurrence is defined as the appearance of newly developed contrast-enhanced lesions on magnetic resonance (MR) images [35]. However, there is no definition for progressive nonenhanced lesions [28]. In this study, progressive and symptomatic new nonenhanced lesions were considered a recurrence. If nonenhanced lesions were identified as progressive after follow-up, the scan at which these changes first appeared during the retrospective review was considered the first scan with progression [35]. One certified neurosurgeon (M.K., 28 years of experience in neurosurgery), who was not blinded either to clinical information or hypothesis, and one certified neuroradiologist (Y.M., 11 years of experience in neuroradiology), who was blinded to clinical information but not to hypothesis, independently accessed the presence or absence of recurrent lesions at DN on T2WI, FLAIR, or Gd-T1WI. Then, M.K., Y.M., and one certified neuroradiologist (E.Y., 8 years of experience in neuroradiology), who was blinded to both, independently accessed the temporal and spatial distribution of abnormal signals on T2WI, FLAIR, or Gd-T1WI at the appearance of lesions and at delayed diagnosis or progression after salvage treatment in cases with recurrence at DN. To estimate the reliability of the assessment of the distribution of recurrent diseases, interrater reliability for the presence or absence of signal changes at each anatomical site was analyzed with the Fleiss kappa statistic [33] using R version 4.3.1 software (R Foundation, Vienna, Austria). Based on a previous report, the ranges are 0.0–0.20 for slight, 0.21–0.40 for fair, 0.41–0.60 for moderate, 0.61–0.80 for substantial, and 0.81–1.0 for almost perfect agreement [17]. Finally, the presence or absence of lesions in each anatomical location was decided by consulting between M.K., Y.M., and E.Y.

Additionally, nonlocal recurrence was classified into LMD and distant intraparenchymal recurrence (DIR) to elucidate the mechanism of recurrence in the DN. LMD was defined as linear or nodular leptomeningeal contrast enhancement along the contours of the gyri, sulcus, cistern, and ventricular wall [2]. DIR was defined as a new intraparenchymal lesion at a site that is not contiguous with the initial tumor location on T2-weighted MR images (T2WI) [30].

### Tractography for depicting the DRTC pathway

Diffusion tensor images (DTI) were acquired using a single-shot spin-echo echo-planar sequence with the following parameters: *b*, 800 s/mm^2^; 15 directions; TR/TE, 5601/63 ms; slice thickness, 3 mm; FOV, 224 × 224 mm; matrix size, 128 × 126; and 50 transverse slices. DTI tractography was performed using a workstation with Brainlab Elements® (Brainlab, Munich, Germany). Two methods of tractography were used. First, a single voxel of interest (VOI) was set in the DN on preoperative gadolinium-enhanced T1-weighted images (Gd-T1WI) fused with DTI. The minimum streamline length was 60 mm, and the fractional anisotropy cutoff was 0.15. Alternatively, DRTC pathway tractography was performed with modifications [11] to detect DRTC pathways that connect the DN with the contralateral thalamus and cortex. The DN and gray matter were segmented automatically, and the contralateral frontal cortex was manually segmented from the latter. Then, seed and target VOIs were set on the DN and contralateral frontal cortex for DRTC pathway tractography. The minimum streamline length was 150 mm, and the fractional anisotropy cutoff was 0.10.

## Results

### Patient background

**Fig. 1:**
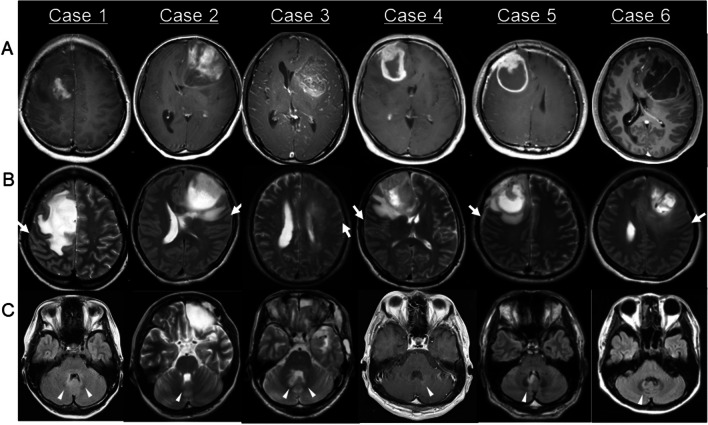
Cases of supratentorial astrocytoma, isocitrate dehydrogenase (*IDH*)-mutant, grade 3 (case 1) and grade 4 (case 2), and glioblastoma, *IDH*-wild type with recurrence in the cerebellar dentate nucleus (DN). **A** Gadolinium-enhanced T1-weighted magnetic resonance (MR) images (Gd-T1WI) at the initial presentation showing the primary tumor in the frontal lobe in all cases. **B** T2-weighted MR images (T2WI) at the initial presentation showing the involvement of the hyperintense area in the premotor area in all cases. Arrows indicate the central sulcus. **C** Fluid-attenuated inversion recovery (FLAIR) (cases 1, 5, and 6) images, T2WI (cases 2 and 3), and Gd-T1WI (case 4) 66, 12, 4, 11, 5, and 5 months after initial tumor resection showing recurrence at the newly developed hyperintense lesions on FLAIR or T2WI or enhanced lesion on Gd-T1WI in the DN and its hilus (arrowheads)

**Fig. 2:**
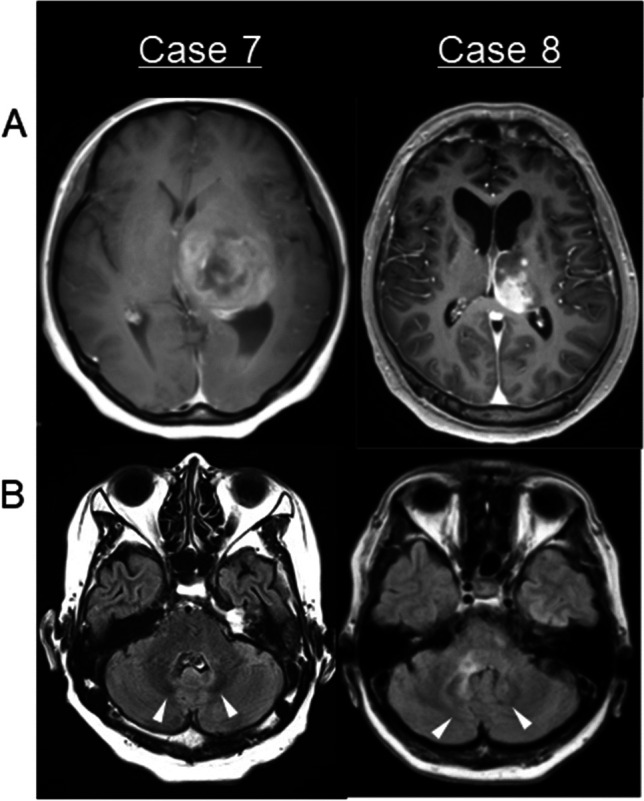
Cases of thalamic diffuse midline glioma, H3 K27-altered (case 7) and glioblastoma (case 8) with recurrence in the cerebellar DN. **A** Gd-T1WI at the initial presentation showing the enhanced area in the contralateral DN. **B** FLAIR 6 and 7 months after tumor resection showing recurrence at the DN and its hilus (arrowhead)

A total of 380 patients with astrocytoma, oligodendroglioma, GB, and thalamic DMG were included in this study. The tumors were located in the frontal lobe in 163 patients, temporal lobe in 115, parietal lobe in 51, occipital lobe in 9, insula in 23, corpus callosum in 3, basal ganglia in 3, and thalamus in 13. Of the 380 patients, two reviewers found 8 (2.1%) identical cases with DN lesions at first recurrence. Table 1 shows the data of the eight patients. Age ranged from 34 to 75 years (median 38 years). Of the eight patients, four were males, and four were females. Among them, two were diagnosed with astrocytoma, grades 3 and 4, five were diagnosed with GB, and one was diagnosed with DMG. The primary lesions were exclusively located in the frontal lobe in six patients (Fig. 1A, B) and the thalamus in two patients (Fig. 2A). In total, recurrence in the DN developed in 8 (7.4%) of 108 patients with high-grade astrocytoma, GB, or DMG at the frontal lobe or thalamus, whereas no other histological types or sites showed recurrence in the DN (Table 2). Of the eight patients, two underwent gross total resection of the enhanced lesion, and six had residual enhanced or nonenhanced lesions. Seven patients received radiotherapy and chemotherapy with temozolomide or nimustine hydrochloride, whereas case 6 was treated with only radiation therapy due to the myelofibrosis. Additionally, hyperintense lesions in the premotor area were noted in all cases with frontal lobe lesions (Fig. 1).

**Table 1:** Clinical characteristics and background of cases with the recurrence in the cerebellar dentate nucleus

Case no	Age/sex	Histological diagnosis	Tumor location	Resection	Initial treatment	Time to recurrence in the DN from initial resection of the tumor (months)	Symptoms at the appearance of recurrence in the DN	Delayed diagnosis after the appearance of recurrent lesions	Side of recurrence in the DN to the primary lesion	Simultaneous recurrence at the primary site	Treatment for recurrence	Further progression site at the last follow-up	Survival after the appearance of cerebellar lesions (months)
1	37/F	Astrocytoma, *IDH*-mutant, grade 3	Rt frontal lobe	Non-GTR	LBI (72 Gy/40 Fr) ACNU	66	None	Yes (24 months)	Bilateral	No	LBI^*^ (60 Gy/30 Fr) TMZ BEV	ST	49 (Dead)
2	34/F	Astrocytoma, *IDH*-mutant, grade 4	Lt frontal lobe	Non-GTR	LBI (60 Gy/30 Fr) ACNU	12	None	Yes (2 months)	Contralateral	Yes	TMZ BEV LBI^*^ (49.5 Gy/15 Fr)	NE	13 (Dead)
3	75/M	Glioblastoma, *IDH*-wild type	Lt frontal lobe and insula	Non-GTR	LBI 60 Gy (60 Gy/30 Fr) TMZ	4	Dysphagia and ataxia	No	Bilateral	Yes	BEV	NE	1 (Dead)
4	40/M	Glioblastoma, *IDH*-wild type	Rt frontal lobe	GTR	LBI 60 Gy (60 Gy/30 Fr) TMZ	11	Ataxia	No	Contralateral	Yes	BEV	IT	2 (Dead)
5	37/M	Glioblastoma, *IDH*-wild type	Rt frontal lobe	GTR	LBI 60 Gy (60 Gy/30 Fr) TMZ	5	Nausea	No	Ipsilateral	No	BEV LBI^*^ (54 Gy/27 Fr)	ST and IT	13 (Dead)
6	35/F	Glioblastoma, *IDH*-wild type	Lt frontal lobe	Non-GTR	LBI 60 Gy (60 Gy/30 Fr)	5	Nausea and ataxia	No	Contralateral	No	LBI^*^ (40 Gy/15 Fr)	ST and IT	5^**^ (Alive)
7	51/F	DMG	Lt thalamus	Non-GTR	LBI 60 Gy (60 Gy/30 Fr) TMZ	6	Nausea	No	Bilateral	No	BEV	ST and IT	5 (Dead)
8	38/M	Glioblastoma, *IDH*-wild type	Lt thalamus	Non-GTR	LBI 50 Gy TMZ TTFields	7	None	Yes (3 months)	Bilateral	No	BEV	NE	6 (Dead)

*M* male, *F* female, *IDH* isocitrate dehydrogenase, *DMG* diffuse midline glioma, H3 K27-altered, *Rt* right, *Lt* left, *GTR* gross total resection of enhanced lesion in the case with glioblastoma and of non-enhanced lesion in that of other histological types, *LBI* local brain irradiation, *Fr* fractions, *ACNU* nimustine hydrochloride, *TMZ* temozolomide, *TTFields* tumor treating fields, *DN* dentate nucleus, *BEV* bevacizumab, *ST* supratentorial lesion, *IT* infratentorial lesion, *NE* not estimated

^*^Radiation to the recurrence outside the initial radiation field

^**^Alive with active disease

**Table 2:** Interrater reliability of the distribution of recurrent lesions in each anatomical site

Anatomical site	Fleiss kappa statistics
DN	0.67
SCP	0.70
Tectum	0.55
Tegmentum	0.49
RN	0.48
Thalamus	0.60
IC	0.41
Premoter	0.51
CV	0.51
CH	0.70
DP	0.47

*DN* dentate nucleus, *SCP* superior cerebellar peduncle, *RN* red nucleus, *IC* internal capsule, *CV* cerebellar vermis, *CH* cerebellar hemisphere, *DP* dorsal pons

To elucidate the frequency of the recurrence at DN among nonlocal failures, the pattern of nonlocal failure in frontal and thalamic primary tumors was further investigated. To this end, nonlocal failure was classified into LMD and DIR. Among the 163 frontal lobe and 13 thalamic tumor cases in this study, LMD was found in 7 and 2 cases, and DIR was found in 13 and 5 cases, respectively. DIR from frontal lobe tumors developed in the DN in 6 (46%) cases, contralateral cerebrum without DN involvement in 3 (23%), brainstem in 2 (15%), cerebellar vermis (CV) without DN involvement in 1 (8%), and cerebellar hemisphere (CH) in 1 (8%). DIR from thalamic tumors developed in the DN in 2 (40%), CH without DN involvement in 1 (20%), CV and hemisphere without DN involvement in 1 (20%), and temporal lobe in 1 (20%). These results showed that the DN was the most frequent site of DIR in frontal lobe and thalamic tumors.

### Diagnosis and concomitant symptoms of DN lesions

The time from the initial treatment to the appearance of abnormal signals ranged from 4 to 66 months (median 7 months) and was longer in cases of astrocytoma than in cases of GB and DMG (Table 1). The appearance of abnormal signals preceded the diagnosis and onset of new symptoms in two cases of astrocytoma (cases 1 and 2) and one case of thalamic GB (case 8). The diagnosis was made with a delay of 2, 3, and 24 months from the appearance of abnormal signals (Table 1). In four cases of GB (cases 3–6) and one case of DMG (case 7), recurrence was detected within one month after the onset of new symptoms, such as nausea and ataxia (Table 1).

### Distribution and extension of recurrent lesions

**Fig. 3:**
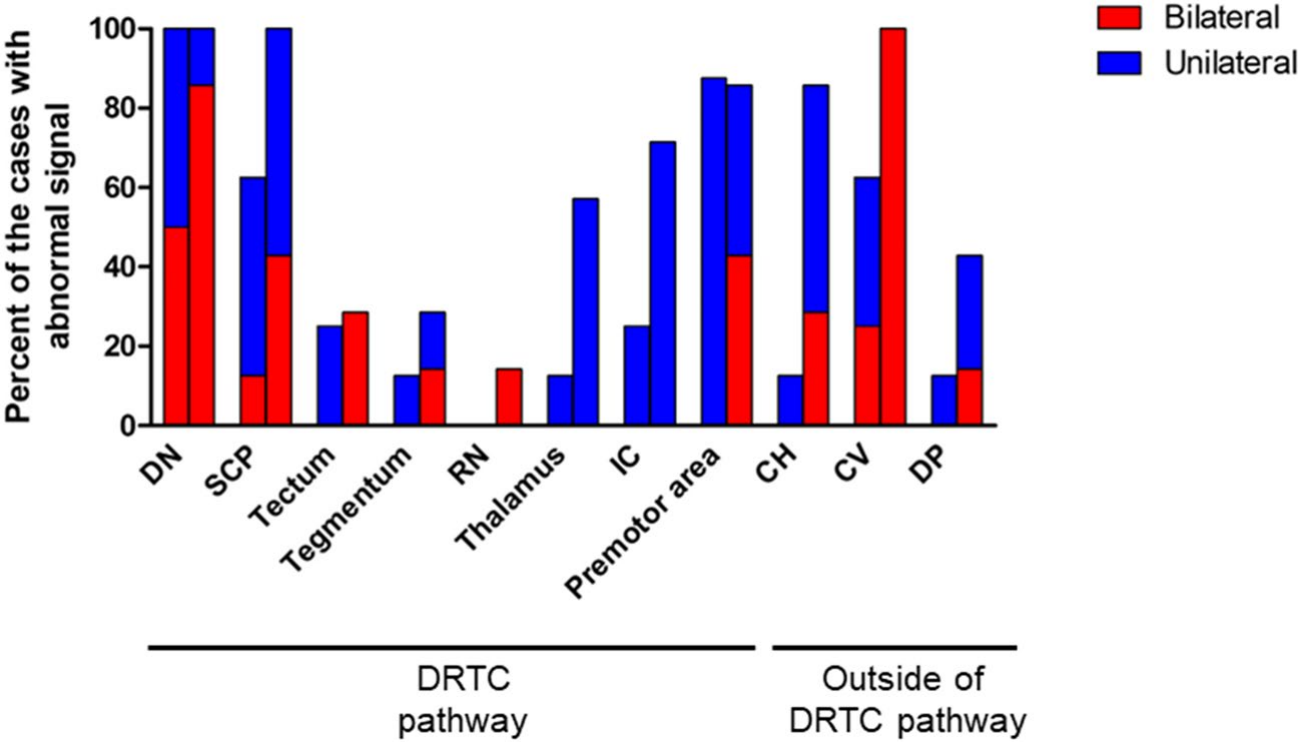
The proportion of cases with the abnormal signal at the time of the appearance of the lesion in eight cases (left bar graph) and at delayed diagnosis or progression after salvage treatment (right bar graph) in seven cases at each anatomical site in and outside the dentato-rubro-thalamo-cortical (DRTC) pathway. Red and blue indicate bilateral and unilateral lesions, respectively. DN and SCP lesions became bilateral lesions, and abnormal signals extended to the tectum, tegmentum, RN, and IC along the DRTC pathway and extended to CV, CH, and DP, which were outside of the DRTC pathway during delayed diagnosis or salvage treatment. *DN* dentate nucleus, *SCP* superior cerebellar peduncle, *RN* red nucleus, *IC* internal capsule, *CV* cerebellar vermis, *CH* cerebellar hemisphere, *DP* dorsal pons, *DRTC* dentato-rubro-thalamo-cortical pathway

**Fig. 4:**
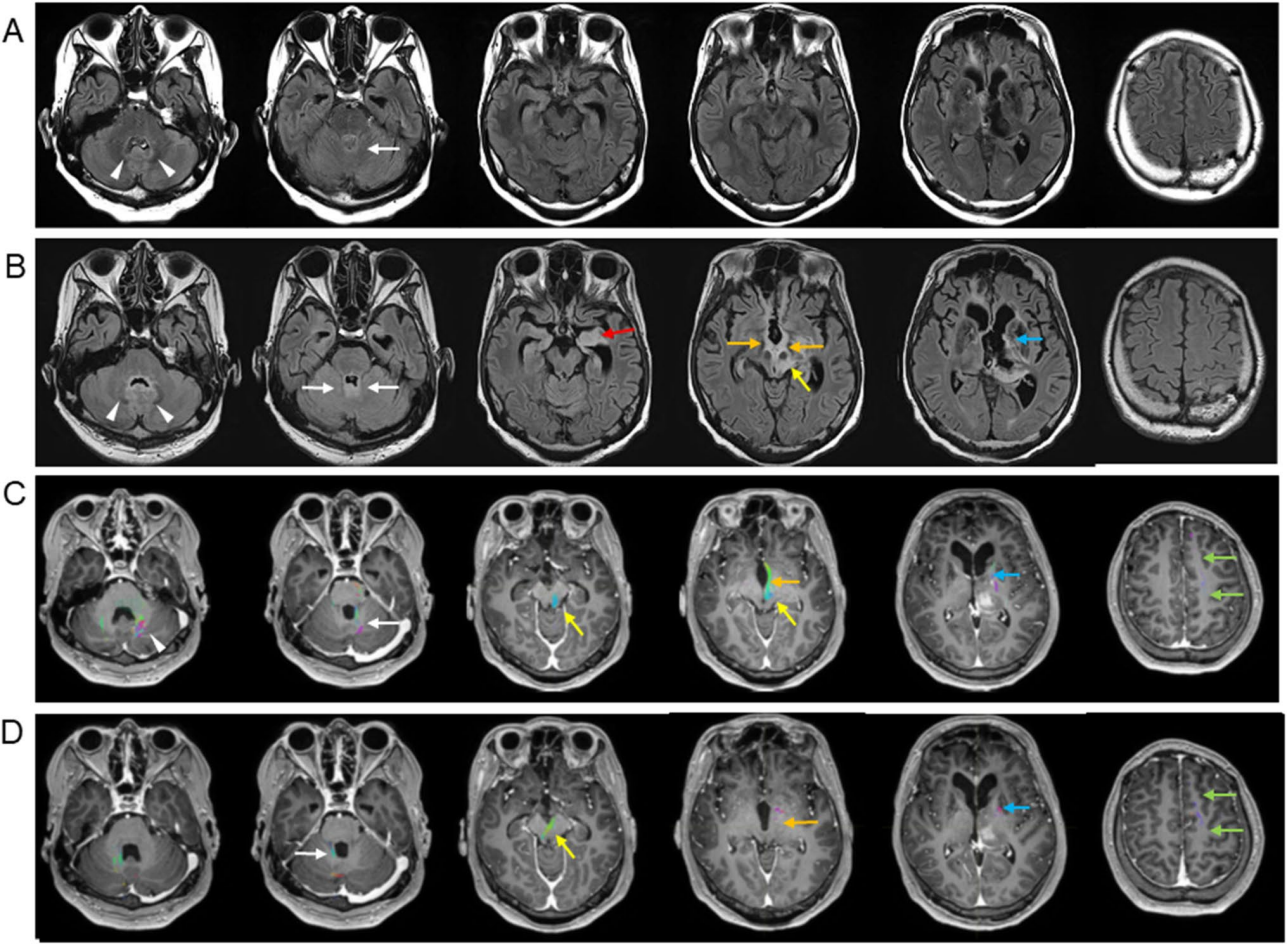
The pattern of further progression after recurrence in the DN on FLAIR images in case 8 with left thalamic glioblastoma, *IDH*-wild type. FLAIR images at the time of the appearance of abnormal signals (**A**) and at delayed diagnosis after 3 months (**B**). For delayed diagnosis, the lesion at the hilus of the DN (arrowhead) and SCP enlarged (white arrow). Recurrence at the primary site and abnormal signals in the tectum and tegmentum, including the red nucleus (RN) (yellow arrow), internal capsule (blue arrow), mammillary body (orange arrows), and amygdala (red arrow), were noted. Preoperative fiber tracking fused with the Gd-T1WI from the left DN (**C**) shows that the tract from the DN (arrowhead) runs through the SCP (white arrow), tectum, and tegmentum, the RN (yellow arrows) and internal capsule (blue arrow), and ends in the frontal lobe (green arrows). Alternatively, preoperative fiber tracking, fused with Gd-T1WI from the right DN to the left frontal cortex (**D**), was run through the SCP (white arrow), tectum, and crossed the tegmentum. It connected the left RN (yellow arrow) and internal capsule (blue arrow) and ended in the frontal lobe (green arrows). Tractography from a single VOI set in the right DN (**C**) and that from seed VOI to target VOI were demonstrated in **C** and **D**, respectively

We tested the hypothesis that recurrence in the DN is a result of infiltration along the DRTC pathway by reviewing the temporal and spatial distribution of recurrent lesions and comparing the distribution of the primary lesion to the DRTC pathway on tractography. The distribution of the lesions was reviewed by three reviewers at the time of the appearance of abnormal signals and further progression. Interrater reliability for accessing the distribution of recurrent lesions was analyzed using Fleiss kappa statistics at each anatomical site (Table 2). Kappa statistics ranged from 0.41 in the internal capsule to 0.70 in the SCP and CH, and the diagnosis of the distribution of recurrent lesions had moderate-to-substantial agreement (Table 2). Three reviewers reached a consensus on the distribution of recurrent lesions at the time of their appearance and progression through consultation. At the time of the appearance of abnormal signals, seven cases (cases 1–3, 5, 6–8) showed hyperintense lesions in the DN and its hilus on fluid-attenuated inversion recovery (FLAIR) MR images or T2WI, and one case (case 4) showed a slightly enhanced lesion at the hilus of the DN on Gd-T1WI without abnormal signals on T2WI (Figs. 1C, 2B, and 4 and Supplemental Fig. 1A). DN lesions were located on the contralateral side of the primary frontal lobe or thalamic lesions in three cases, the ipsilateral side in one case, and both sides in four cases (Table 1 and Figs. 1, 2, and 3). Simultaneous abnormal signals were frequently observed in the SCP, CV, and premotor area (Figs. 3 and 4A). Additionally, local recurrence of the primary lesions was observed in three cases (Table 1). The pattern of further progression was reviewed in three cases in which lesions progressed due to delayed diagnosis and in four cases in which lesions progressed after various salvage treatments to clarify the pattern of progression after recurrence. One case (case 3) was not followed up because of the rapid progression of the disease. Seven cases experienced progression of the DN lesion. Histological finding was verified only in case 1 because it seemed to be an atypical distant recurrence of the glioma. Histological diagnosis of the DN lesion in this case was astrocytoma, IDH mutant, grade 3 that was identical to primary tumor. We did not verify the histological diagnosis in all other cases due to their rapid progression. The number of cases with abnormal signals in the DN, SCP, tectum, tegmentum, RN, thalamus, internal capsule, and premotor area is shown in Fig. 3. The lesions extended to bilateral DN and SCP, and to tectum, tegmentum, thalamus, and internal capsule. In particular, SCP lesions extending from the DN were found in all cases (Fig. 3). Furthermore, the lesions progressed to the CH, dorsal pons, CV, mammillary body, and amygdala (Figs. 3 and 4B and Supplemental Fig. 1B). The MR images at the last follow-up showed no evidence of LMD. These findings indicate that the recurrence in DN was a result of infiltration along the neural network rather than dissemination through the CSF.

Next, the relationship between the DRTC pathway and preoperative lesions was investigated in four cases. The DRTC pathway from the ipsilateral and contralateral DN, in which distant recurrence developed to primary lesions, to the affected thalamus could be tracked in two thalamic gliomas. The tract from DN runs through the ipsilateral SCP and tectum, crosses or does not cross the tegmentum, runs through the left RN and internal capsule, and ends in the left frontal lobe (Fig. 4C, D). The fibers from DN are also connected to the mammillary body (Fig. 4C). The distribution of fibers from DN on fiber tracking corresponded to the recurrence site (Fig. 4B–D). However, it was difficult to track the DRTC pathway from the DN in two cases with frontal lobe glioblastoma because of the mass effect of the enhanced lesions. Instead, fiber tracking from the right DN was connected to the contralateral frontal lobe along the DRTC pathway. Recurrence was located at the site corresponding to the SCP on the DRTC pathway (Supplemental Fig. 1B and 1C) and at the symmetrical site of the left internal capsule on the DRTC pathway (Supplemental Fig. 1B and 1C).

These results and the finding of fiber tracking from the DN showed that the recurrence in the DN was strongly associated with the DRTC pathway and that the lesions extended beyond the DRTC pathway at further progression.

### Treatment and prognosis for recurrence

Although bevacizumab, temozolomide, and local irradiation were administered for recurrent lesions, 5 of 6 patients with GB and DMG died within 1–13 months (median 5 months) after the appearance of abnormal signals due to the progression of supratentorial and/or infratentorial recurrent lesions (Table 1).

## Discussion

In this study, distant recurrence in the DN was observed in 2.1% of the patients with supratentorial adult diffuse gliomas and thalamic DMG. The recurrence developed in 7.4% of the patients with astrocytoma, grades 3 and 4, GB, and DMG at the frontal lobe or thalamus. This recurrence pattern was characteristic of high-grade astrocytoma, GB, and DMG in the frontal lobe or thalamus, and the DN was the most frequent site of DIR in cases of frontal lobe and thalamic tumors. Clinically, the symptoms were preceded by the appearance of abnormal findings in three cases, while the diagnosis was established within 1 month after the onset of new symptoms in cases of GB and thalamic DMG. The pattern of further progression revealed abnormal findings extending from the DN and SCP to the anatomical structure along the DRTC pathway without LMD. These findings indicate that recurrence in the DN developed through infiltration along the DRTC pathway, not dissemination through the CSF.

The diagnosis of recurrence in the DN has been reported to be sometimes delayed because of the absence of abnormal findings on MR images despite intractable symptoms of nausea [7]. This study showed that the diagnosis was sometimes delayed because of the absence of symptoms despite the abnormal findings on MR images. In contrast to the cases with GB, in which diagnosis was established immediately due to the severe symptoms, two cases of astrocytoma had no symptoms at the time of the appearance of the hyperintense area in the DN, and it took 2 and 24 months to diagnose the DN lesion. Based on these findings, attention should be paid to the findings in the DN and SCP during the follow-up of astrocytoma, even though they were asymptomatic.

Kawauchi et al. reported a case series of cerebellar recurrence from supratentorial lesions and described that the recurrence might develop along the DRTC pathway [14]. In this study, we hypothesized that recurrence in the DN developed along the DRTC pathway based on the anatomical background of the DN. This study showed some new findings. First, the predominant tumor location of the primary tumor was clarified. To the best of our knowledge, 10 cases of adult diffuse gliomas with recurrence in the DN have been reported in the literature [4, 7, 12, 14]. Primary lesions in these cases were located at the frontal lobe in seven cases, thalamus in one case, and temporal lobe in one case [4, 7, 14]. Additionally, a case of histologically diagnosed anaplastic oligodendroglioma was previously reported, in which the recurrence in the DN developed 12 months after resection [12]. MR images and histological and molecular findings were reviewed and revealed astrocytoma, *IDH* mutant, grade 4, at the frontal lobe as well as the temporal lobe and insula. Based on these findings, distant recurrence in the DN is a specific failure pattern in cases with diffuse gliomas in the frontal lobe and thalamus. The mechanism by which recurrence in the DN predominantly occurs in patients with frontal lobe lesions can be explained by the fact that the frontal lobe is the main projection site in the DRTC pathway [15]. In a previous study, an analysis using DTI in 39 healthy subjects revealed that the DRTC pathway projects widely to the frontal lobe, with 30–80% of fibers projecting to Brodmann’s area 6 [11]. This finding was consistent with that reported in our study, which showed that the premotor area was involved in all cases with frontal lobe lesions. Second, lesions occurred not only on the contralateral side of the frontal lobe but also on the ipsilateral side. The experiment with retrograde transneuronal transport in primate and multifiber probabilistic models demonstrated dominant connections to the contralateral frontal lobe [23], and these connections were also demonstrated in tractography [11]. Tractography in humans and analysis of retrograde tracer transport in monkeys also revealed that fibers from DN also connect to the ipsilateral thalamus and frontal lobe [3, 15, 18, 21, 23]. Based on previous reports [4, 7, 12, 14] and our results, DN lesions developed on the contralateral side in nine, the ipsilateral side in two, and both sides in eight. This result reflects the connections of DN to the contralateral and ipsilateral thalamus and the frontal lobe. Third, abnormal signals first appeared in the DN, which is the most distal part of the DRTC pathway, away from the frontal lobe and thalamus. One possible mechanism is that these regions did not receive adequate radiation dosage despite infiltration along the DRTC pathway. Similarly, 10% of glioblastoma cases had out-field recurrence [25]. Another possible explanation is that the DN can be a favorable environment for tumor growth. Recently, the formation of a network of neurons has been reported to be associated with tumor growth [33]. We presume that the network in the DN may play an important role in distant recurrence.

Salvage irradiation to the recurrence in the DN can be a safe and promising approach because the posterior fossa is not included in the irradiation field for the initial lesion, especially in frontal lobe lesions. In this study, four patients with GB and DMG treated with bevacizumab alone died within 5 months, whereas two patients treated with irradiation of the recurrence in the cerebellum and brainstem survived for more than 6 months. However, the supratentorial and infratentorial lesions eventually became uncontrollable in these two cases. This is a limitation of local treatment, which focuses only on cerebellar and brainstem lesions in cases with extensive infiltration along the DRTC pathway. Future development of a treatment based on the evolutionary pathway may improve prognosis.

This study has some limitations. First, although the results were based on 380 cases of glioma, the number of cases analyzed was small. Thus, further studies using the data of large-scale registry projects or prospective clinical studies are needed to validate our results. Second, this was a retrospective study, and some neurological symptoms at the time of the appearance of abnormal signals might have been overlooked. Third, we cannot provide direct evidence due to the lack of estimation by DTI at recurrence. Instead, we attempted to examine fiber tracking from preoperative images. Although it was difficult to track the crossing fibers from the DN to the contralateral cerebrum and compressed fibers in the presence of a frontal enhanced lesion [23], the relationship between the ipsilateral DRTC pathway or the DRTC pathway without glioma and the anatomical site of recurrence strongly suggested that recurrence developed along with the DRTC pathway. Fourth, histological diagnosis of the DN lesion was verified because of rapid progression and concern regarding neurological complication of the tissue diagnosis. To clarify this issue, autopsy can provide important clues for our hypothesis.

In conclusion, distant recurrence in the DN is common in malignant gliomas of the frontal lobe and thalamus. Thus, attention should be paid to the cerebellar and brainstem symptoms and the abnormal findings of the DN and SCP on MR images. Distant recurrence in the DN from supratentorial glioma was suggested to be a result of dissemination through the CSF. However, our results showed that it developed as a result of infiltration along the DRTC pathway.

## Supplementary Information

Below is the link to the electronic supplementary material.

Supplementary file1 (DOCX 607 KB)

## Abbreviations

CH: Cerebellar hemisphere
CSF: Cerebrospinal fluid
CV: Cerebellar vermis
DIR: Distant intraparenchymal recurrence
DMG: Diffuse midline gliomas H3 K27-altered
DN: Dentate nucleus
DP: Dorsal pons
DRTC: Dentato-rubro-thalamo-cortical
DTI: Diffusion tensor images
FLAIR: Fluid-attenuated inversion recovery
GB: Glioblastoma, *IDH*-wild type
Gd-T1WI: Gadolinium-enhanced T1-weighted images
IC: Internal capsule
IDH: Isocitrate dehydrogenase
LMD: Leptomeningeal dissemination
MR: Magnetic resonance
RN: Red nucleus
SCP: Superior cerebellar peduncle
T2WI: T2-weighted MR images
VOI: Voxel of interest
