# Supplementary material for: Distant recurrence in the cerebellar dentate nucleus through the dentato-rubro-thalamo-cortical pathway in supratentorial glioma cases
Source: Acta Neurochir (Wien). 2024 Feb 14;166(1):83. doi: 10.1007/s00701-024-05981-8 (PMC10867057; doi:10.1007/s00701-024-05981-8)
Supplement: Supplementary file 1 — Supplementary file1 (DOCX 607 KB) [file 701_2024_5981_MOESM1_ESM.docx]

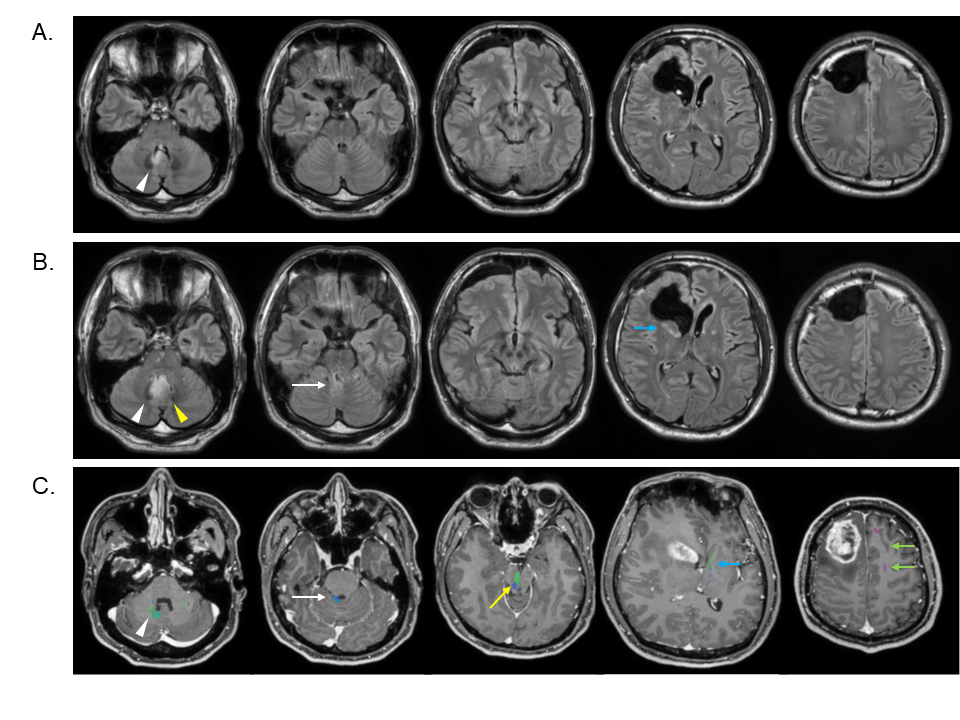
**Supplemental Fig. 1.**

The pattern of further progression after recurrence in the dentate nucleus (DN) on fluid-attenuated inversion recovery (FLAIR) images in case 5 with right frontal lobe glioblastoma, isocitrate dehydrogenase-wildtype. FLAIR images at the time of the appearance of abnormal signals (A) and at progression after salvage treatment with bevacizumab (B). The lesion at the hilus of the DN enlarged (white arrowhead) and extended to the DN, cerebellar vermis (yellow arrowhead), superior cerebellar peduncle (SCP) (white arrow), and internal capsule and thalamus (blue arrow). Preoperative fiber tracking fused with gadolinium-enhanced T1-weighted magnetic resonance images from the right DN (C) shows that the tract from the DN (white arrowhead) runs through the SCP (white arrow), tectum, and tegmentum, including the RN (yellow arrow) and left internal capsule (blue arrow), and ends in the left frontal lobe (green arrows). Progression after first recurrence in panel B developed along with ipsilateral SCP and ipsilateral internal capsule, which was the symmetrical site on the left internal capsule on the dentato-rubro-thalamo-cortical (DRTC) pathway. The primary tumor at the right frontal lobe was located on the symmetrical site of the left frontal lobe on the DRTC pathway.
